# Dental Pulp Stem Cell-Derived, Scaffold-Free Constructs for Bone Regeneration

**DOI:** 10.3390/ijms19071846

**Published:** 2018-06-22

**Authors:** Fukushima Tatsuhiro, Tatehara Seiko, Takebe Yusuke, Tokuyama-Toda Reiko, Satomura Kazuhito

**Affiliations:** Department of Oral Medicine and Stomatology, School of Dental Medicine, Tsurumi University, 2-1-3 Tsurumi, Tsrumi-ku, Yokohama 230-8501, Japan; fukushima-tatsuhiro@tsurumi-u.ac.jp (F.T.); takebe-yusuke@tsurumi-u.ac.jp (T.Y.); tokuyama-r@tsurumi-u.ac.jp (T.-T.R.); satomura-k@tsurumi-u.ac.jp (S.K.)

**Keywords:** bone induction, bone regeneration, cell sheet, dental pulp stem cell, scaffold-free, three-dimensional culture, tissue engineering construct

## Abstract

In the present study, a scaffold-free tissue construct was developed as an approach for the regeneration of tissue defects, which produced good outcomes. We fabricated a scaffold-free tissue construct from human dental pulp stem cells (hDPSCs construct), and examined the characteristics of the construct. For its fabrication, basal sheets prepared by 4-week hDPSCs culturing were subjected to 1-week three-dimensional culture, with or without osteogenic induction, whereas hDPSC sheets (control) were fabricated by 1-week culturing of basal sheets on monolayer culture. The hDPSC constructs formed a spherical structure and calcified matrix that are absent in the control. The expression levels for bone-related genes in the hDPSC constructs were significantly upregulated compared with those in the control. Moreover, the hDPSC constructs with osteogenic induction had a higher degree of calcified matrix formation, and higher expression levels for bone-related genes, than those for the hDPSC constructs without osteogenic induction. These results suggest that the hDPSC constructs with osteogenic induction are composed of cells and extracellular and calcified matrices, and that they can be a possible scaffold-free material for bone regeneration.

## 1. Introduction

Autologous bone graft is the conventional gold standard treatment method for alveolar atrophy and jawbone defect [1]. However, bone graft may not sufficiently achieve the therapeutic goal, due to the invasiveness of bone collection and/or limitations in the amount of bone collected from a donor site [2,3]. Currently available biomaterials, including hydroxyapatite and β-tricalcium phosphate, have been improved and used in clinical practice. However, they have some concerns, such as the time-consuming process for bone replacement, inflammatory reactions that can occur during biomaterial degradation, and bacterial infections [4,5].

In recent years, the technology of designing and constructing artificial tissue-engineered constructs (TECs) that are similar to those found in living organisms has been developed in regenerative medicine and tissue engineering. These constructs can be applied to regenerate a defective tissue or to regain dysfunctional organs. In TEC fabrication methods, biodegradable scaffolds, such as collagen, gelatin, and lactic acid–glycolic acid copolymer, have been used to support seed and culture cells [6]; this can facilitate cell proliferation and differentiation through cell–cell and cell–extracellular matrix interactions [7]. These biomaterials allow the development of mutual associations among cells, extracellular matrices, and growth factors to mimic in vivo cellular reactions, because they permit cell culturing under a 3D environment. However, the cells do not migrate to the inside of the biomaterials [8]. Moreover, biodegradable materials are at a high risk of causing bacteria infection, and immune reactions during degradation [9]. To avoid these problems, various TECs without scaffolds are being developed. A cell sheet fabricated using a dish with a temperature-responsive polymer is a representative scaffold-free TEC [10]. This sheet can be grafted without suturing because extracellular matrices retained in the sheet allow its direct adherence to an organ. Cell sheets have been applied in clinical practice, including myocardial [11], esophageal and gastric mucosal [12], and corneal regeneration [13], and have provided good therapeutic effects. However, regenerative treatment-related issues using cell sheet engineering include surgical invasion due to the collection of functional cells from healthy tissues, and unsuccessful garnering of good-quality functional cells. Thus, many researchers have successfully reported tissue engineering techniques using mesenchymal stem cells as the cell source [14,15,16]. Reports have demonstrated that such TECs provide safety and efficacy over a longer time, even in vivo, compared with those fabricated using scaffolds [17].

Mesenchymal stem cells present in dental pulp tissue [18,19], which are also known as dental pulp stem cells (DPSCs), have a high capacity for proliferation and differentiation into bone, cartilage, muscle, and adipose tissue. DPSCs can be collected from an extracted tooth (medical waste) without invasion, and can be cryopreserved while maintaining their proliferation and differentiation capacities [20,21,22]. Therefore, DPSCs have recently gained attention as a superior cell source for bone/alveolar regeneration, and development of DPSC grafts with biodegradable material and/or apatite has been attempted [23,24]. We speculated that DPSCs are a good cell source for scaffold-free TECs for bone regeneration.

Therefore, we aimed to develop a method of easily fabricating scaffold-free TECs from human DPSCs for bone regeneration. We produced TEC by culturing human DPSC sheets under 3D culture. To use TEC as the novel cell graft therapy for efficient bone regeneration, we examined the conditions that increased the capacity of TECs for osteogenic differentiation and calcified matrix formation, and analyzed the characteristics of TECs.

## 2. Results

### 2.1. Characterization of Human Dental Pulp Stem Cells (hDPSCs)

**Figure 1:**
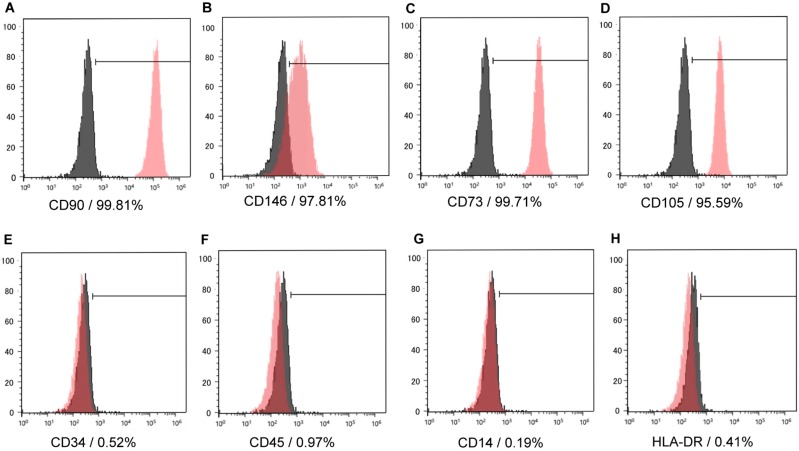
Representative histograms of cell surface markers on isolated cells. The isolated cells were analyzed at passage 3 using a flow cytometer (*n* = 3). The red area shows the histogram for positive cells, and black area indicates isotype controls. The isolated cells were positive for CD90, CD146, CD73, and CD105, which are mesenchymal stem cell markers (**A**–**D**), and negative for CD45, CD34, CD14, and HLA-DR, which are hematopoietic cell markers (**E**–**H**). The red area shows the histogram for positive cells, and black area indicates isotype controls.

We isolated and cultured cells from dental pulp tissue using enzymatic treatment. These cells, which were attached to the culture plate, were a heterogeneous population with polygonal or fibroblast-like spindle shapes. Flow cytometry showed that dental pulp cells (DPCs) were positive for CD90, CD146, CD73, and CD105 as mesenchymal stem cell markers (Figure 1A–D), and negative for CD45, CD34, CD14, and HLA-DR (Figure 1E–H) as hematopoietic stem cell markers, which suggested that the cells were hDPSCs.

### 2.2. Findings of hDPSC Construct

**Figure 2:**
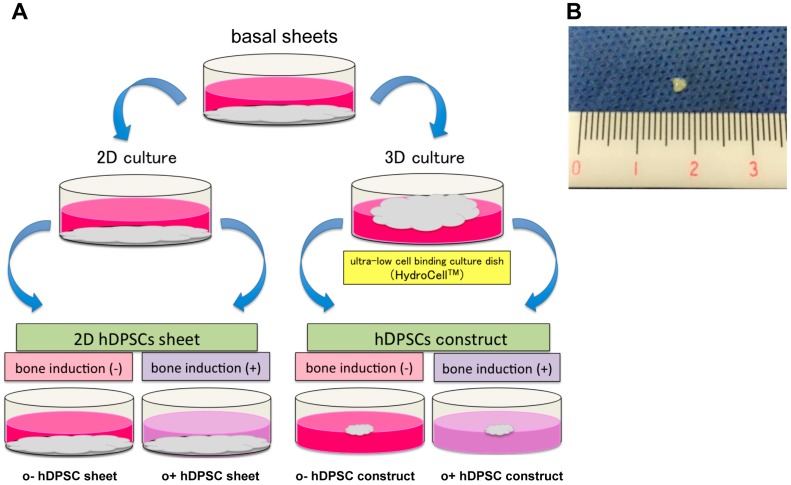
Schematic diagram for preparation of the human dental pulp stem cell (hDPSC) sheets and hDPSC constructs (**A**). Macroscopic of the hDPSC construct (**B**).

To demonstrate the characteristics of hDPSC construct, the basal sheets were produced by culturing hDPSC on cell plates for 4 weeks, and then scraping off the cell monolayer (Figure 2A). These basal sheets were re-plated under four different conditions (Figure 2A). After one week of culture, the constructs exhibited a spherical structure (3–4 mm diameter; Figure 2B). The hDPSC constructs were confirmed to be elastic, and to retain their shape even after grasped with tweezers.

In the control, hDPSC sheets were cultured in the basal (o−hDPSC sheet) and osteogenic (o+hDPSC sheet) media using monolayer culture. Then, the sheets were cultured in super low adherent culture plate (HydroCell™) in basal (o−hDPSC construct) or osteogenic (o+hDPSC construct) medium using three-dimensional (3D) environment culture for a week (Figure 2A). Macroscopic findings of the hDPSC construct exhibiting a spherical structure with a diameter of 3–4 mm (Figure 2B).

### 2.3. Histological Findings

**Figure 3:**
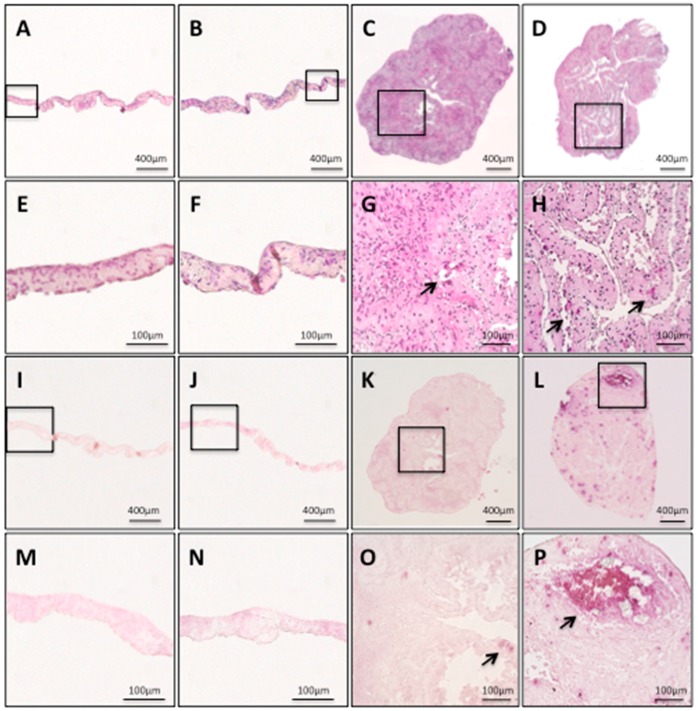
Histological findings of a hDPSC sheet and hDPSC construct. o−hDPSC sheet H&E staining (**A**) ×40; (**E**) magnification, ×200. o+hDPSC sheet H&E staining (**B**) ×40; (**F**) magnification, ×200. o−hDPSC construct H&E staining (**C**) ×40; (**G**) magnification, ×200. o+hDPSC construct H&E staining (**D**) ×40; (**H**) magnification, ×200. o−hDPSC sheet alizarin red S staining (**I**) ×40; (**M**) magnification, ×200. o+hDPSC sheet alizarin red S staining (**J**) ×40; (**N**) magnification, ×200. o−hDPSC construct alizarin red S staining (**K**) ×40; (**O**) magnification, ×200. o+hDPSC construct alizarin red S staining (**L**) ×40; (**P**) magnification, ×200. Black arrows indicate a calcified matrix

The hDPSC sheets and hDPSC constructs were histologically investigated. The hDPSC sheets and hDPSC constructs were histologically investigated. Both hDPSC sheets had sheet structures with some cell layers (Figure 3A,B). The hDPSC sheets showed no calcified matrix formation within the cell sheets, regardless of whether osteogenic induction medium was used (Figure 3E,F); there was no area stained by alizarin red S (Figure 3I,J,M,N), while both hDPSC constructs had a shape like a cellular spheroid, which was composed of extracellular matrix and cells (Figure 3C,D). The findings from the hDPSC constructs revealed calcified matrix formation within the constructs (indicated by arrows in Figure 3G,H), and calcium deposition stained by alizarin red S (Figure 3K,L,O,P). In particular, o+hDPSC construct formed the most calcified matrix (Figure 3L,P).

**Figure 4:**
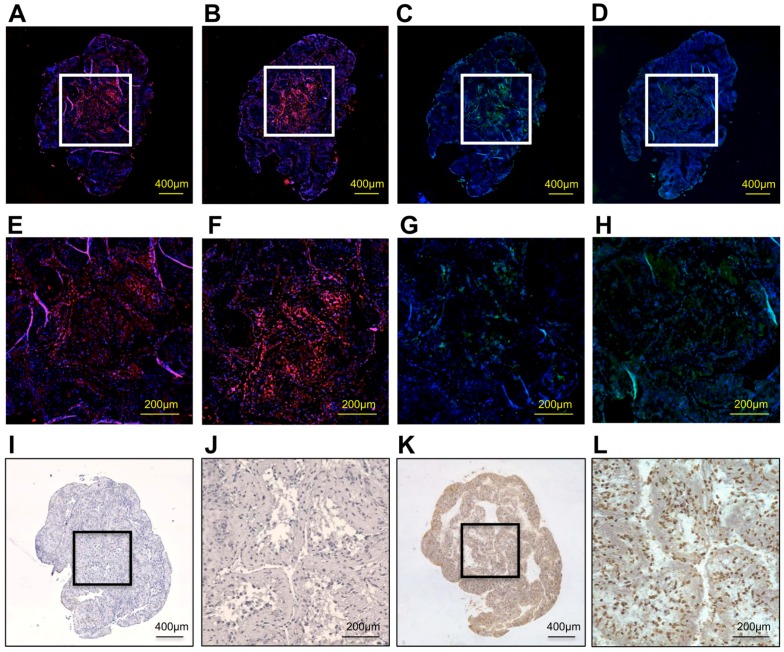
The immunohistological findings of a representative o+hDPSC construct. The o+hDPSC construct expressed osteopontin (red) (**A**) ×40; (**E**) magnification, ×200, bone sialoprotein (red) (**B**) ×40; (**F**) magnification, ×200, osteocalcin (green) (**C**) ×40; (**G**) magnification, ×200 and type1 collagen (green) (**D**) ×40; (**H**) magnification, ×200. In particular, expressions of osteopontin, bone sialoprotein, and osteocalcin were shown in its center, and the expression of type1 collagen was extensively displayed. These bone-related proteins were strongly stained in the o+hDPSC construct. o+hDPSC construct showed no TUNEL-positive cells in its center (**I**) ×40; (**J**) magnification, ×200. A number of TUNEL-positive cells were observed in the positive control group (brown) (**K**) ×40; (**L**) magnification, ×200.

Immunohistological findings showed that a representative o+hDPSC construct expressed the bone-related proteins osteopontin (OPN), bone sialoprotein (BSP), osteocalcin (OCN), and type I collagen (Col 1; Figure 4A–D). Particularly, the expression of OPN, BSP, and OCN was strongly detected in the center of the construct (Figure 4E–G), whereas that of Col 1 was extensively observed throughout the construct (Figure 4H). These bone-related protein factors were strongly stained in the o+hDPSC construct. The o+hDPSC constructs showed no terminal deoxynucleotidyl transferase dUTP nick-end labeling (TUNEL)-positive cells in the center (Figure 4I,J), whereas numerous TUNEL-positive cells were observed in the positive control group (Figure 4K,L).

Both hDPSC sheets had no calcified matrix formation within the sheets, regardless of whether the osteogenic medium was used (Figure 3E,F), and none of the areas were stained by alizarin red S (Figure 3M,N). On the other hand, both hDPSC constructs had numerous formations of calcified matrix, which are indicated by the arrows (Figure 3G,H,O,P).

### 2.4. Expression of Bone-Related Genes and Calcium Content in hDPSC Construct

**Figure 5:**
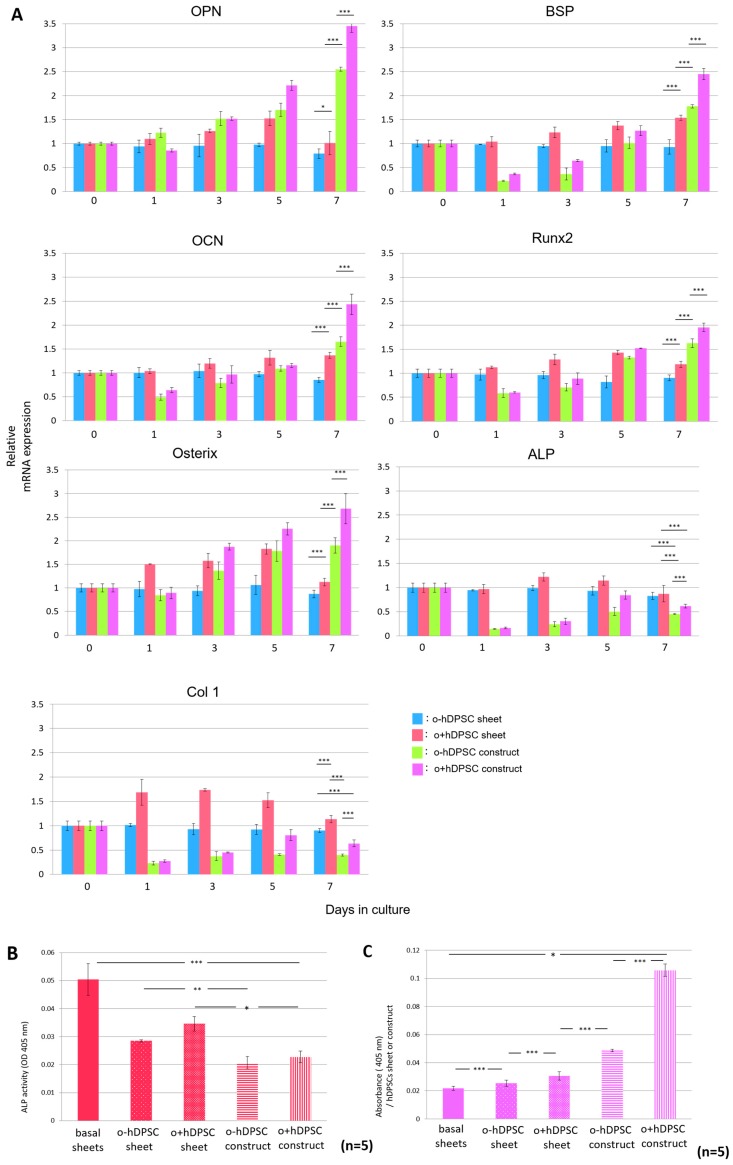
Expression of bone-related genes and calcium content in the hDPSC construct. The time-dependent change of expression of osteogenesis-specific genes in an hDPSC construct (*n* = 3) (**A**) The hDPSC construct exhibited a significantly higher expression of *OPN*, *BSP*, *OCN*, *Runx2*, and *osterix* on day 7 after the initiation of culture. Moreover, 3D culture with bone induction significantly promoted greater expressions of these genes. Furthermore, this culture significantly decreased the expression of *ALP* and *Col1* genes. * *p* < 0.05; *** *p* < 0.001. The ALP activity of a hDPSC sheet and hDPSC construct (*n* = 5) (**B**); The hDPSC construct had significantly decreased ALP activity in comparison with hDPSC sheets. In addition, o+hDPSC construct had significantly decreased ALP activity in comparison with hDPSC sheets. * *p* < 0.05; ** *p* < 0.01; *** *p* < 0.001. The quantity of calcified matrix formed in a hDPSC sheet and hDPSC construct (*n* = 5) (**C**). The quantity of calcified matrix formed in a hDPSC construct was significantly higher than hDPSC sheets. In addition, a hDPSC construct cultured in osteogenic medium formed the highest amount of calcification. * *p* < 0.05; *** *p* < 0.001.

Real-time polymerase chain reaction (PCR) was used to analyze bone-related gene expression over time in each sample cultured in each condition. We analyzed representative samples, as described in the following section. Compared with the hDPSC sheets, the hDPSC constructs exhibited significantly higher *OPN*, *BSP*, *OCN*, *Runx2*, and *osterix* expressions on day 7 after culture initiation. Moreover, o+hDPSC constructs promoted greater expression of these genes, whereas the expression levels of alkaline phosphatase (*ALP*) and *Col1* in the o+hDPSC constructs were significantly lower than that of the hDPSC sheets (Figure 5A). The same analysis was performed for four other samples with similar results. Next, to analyze ALP activity in the hDPSC construct and sheets, ALP activity values and DNA amount of each sample were measured, and the results were comparatively investigated by calculating the ALP activity per unit DNA. After seven days of 3D culture, the ALP activity in hDPSC constructs significantly decreased compared with that in the hDPSC sheets, indicating a similar trend to that observed for *ALP* gene expression (Figure 5B). Lastly, the quantification of alizarin red S staining showed that the ability of formation of calcified matrix in the hDPSC constructs was significantly higher than that in the hDPSC sheets, regardless of whether osteogenic induction was used. Furthermore, o+hDPSC constructs had the highest amount of calcification (Figure 5C).

## 3. Discussion

In this study, we fabricated scaffold-free hDPSC constructs, and demonstrated that they comprised cells, extracellular matrices produced by the cells, and calcified matrices. It is generally considered that extracellular matrices synthesized by the cells in a scaffold-free construct can act as a scaffold. The o+hDPSC construct could be used as an ideal scaffold-free construct for bone regeneration, because it has extracellular matrices expressing bone matrix proteins, as well as calcified matrices. We also showed that the o+hDPSC construct could be fabricated without using any artificial materials, thereby providing a solution to overcome the problems arising from the use of artificial materials. Numerous methods for fabrication of a scaffold-free TEC have been reported. Pellet culture is a method for fabricating a spheroid by centrifuging a large number of cells [25]. However, this method cannot be directly applied to most clinical situations, because of limitations in the mass size of the materials [17]. Therefore, many studies on biofabrication, which is a next-generation tissue engineering technology, have recently reported tissue preparation from cells via engineering, such as using a method to fabricate scaffold-free TECs that do not induce cellular and tissue necrosis [26,27,28]. Specific methods for TEC include the layering of cell sheets prepared using a temperature-responsive culture dish [29], and molding a large number of spheroids using cylindrical glass molds and agarose molds [30,31]. These techniques have enabled the development of large-sized TECs. However, it is difficult to practically apply such TECs to tissue regeneration at present, due to issues concerning the use of special machines for fabrication and complex manufacturing process. Our hDPSC constructs required no special machinery for fabrication; a scaffold-free TEC can easily be constructed by culturing the cells in an ultra-low, cell-binding culture dish. The constructs were also characterized by superior grafting operability because they could easily be held by forceps due to their spherical structure with 3–4 mm diameter. These features make TECs potentially suitable graft materials for clinical application.

Extracellular matrices comprising the o+hDPSC construct expressed bone-related proteins. The study of the expression distribution of these proteins showed that Col 1 was expressed widely from the surface layer of the tissue structure to its center, whereas OPN, BSP, and OCN were expressed in the center of the structure (Figure 4E–H). This tissue structure suggested a possible role of Col 1 as a scaffold of the o+hDPSC construct. Actually, Col 1 is used as a scaffold in conventional tissue engineering [32], and is also known to play an important role in bone tissue regeneration [33]. During the regeneration process, bone is formed through the deposition of calcified matrices on osteoblast-produced Col 1 that acts as a scaffold [34]. Our histological findings suggested that Col 1 was first formed as a frame of the o+hDPSC construct, followed by calcification that initiated from the center. Takewaki et al., fabricated a scaffold-free construct with a similar method as our method using canine bone marrow mesenchymal stem cells in an osteogenic induction medium [14]. They reported that Col 1 was the main component of this scaffold-free construct, and that calcification started from the center. Our o+hDPSC construct structure was consistent with the scaffold-free construct of Takewaki et al., although the origin of the stem cells differed. Moreover, grafting of the scaffold-free construct to a bone defective site led to the promotion of osteogenesis, indicating that the o+hDPSC construct may be used as a scaffold-free graft material with high usefulness for bone regeneration.

Hypoxic conditions in the center of the o+hDPSC construct are likely to be involved with the initiation of osteoblast differentiation, resulting in calcified matrix formation. It was reported that the center of a spheroid fabricated under 3D culture can generally become hypoxic [8]. In addition, another study reported that the culture of DPCs under a hypoxic environment containing 5% O_2_ led to the promotion of differentiation of DPCs into osteoblasts, and formation of calcified matrix [35]. We speculated that the hypoxic conditions at the center of the o+hDPSC construct caused calcified matrix formation via the induction of differentiation into osteoblasts. However, a TUNEL staining assay failed to detect apoptosis at the center of all the o+hDPSC construct samples despite 3D culturing for 7 days (Figure 4J). This could be partially attributed to the cell type; hDPSCs have a very high proliferation capacity, and it has been shown that their proliferation capacity is enhanced in hypoxic conditions [36,37,38]. Therefore, cell death may not have been detected at the center, despite the hypoxic environment. Another likely reason may be the influence of voids (Figure 3D,H) existing in the o+hDPSC construct. In the construct fabricated by Takewaki et al., voids were found inside the construct on day 5 of the 3D culture, and no apoptosis was observed inside the construct during this period. However, the voids disappeared by day 10, after which apoptosis was confirmed. In a construct fabricated by layering human alveolar periosteum-derived cell sheets, voids existed between the sheets during the early stage of layering, but shrank as the culture time elapsed. This phenomenon was also observed in the process of shrinkage of the layered cell sheets, partly due to increased intercellular binding caused by the proliferation of component cells and their extracellular matrix production after the start of the 3D culture [39]. Based on these reports, we speculated that the voids found inside our construct tended to shrink as culture time elapsed, and maintained oxygen and nutrition supplies to the center, to some extent, while inside the construct. In other words, it is likely that the presence of the voids inside the construct contributed to the maintenance of cellular activities at the center.

We noted interesting results regarding the time-dependent expression of bone-related genes inside the hDPSC constructs after initiating the 3D culture. Expression of *OPN*, *BSP*, and *OCN* marker genes found in the middle-to-late stages of the osteoblast differentiation process, was enhanced until day 7 of the culture. The expression of these genes was significantly higher than that of genes observed for the hDPSC sheet (Figure 5A). Culture in the 3D environment substantially contributed to this phenomenon. To some extent, culture in the 3D environment can reproduce the original microenvironment, consisting of cells and extracellular matrices, and promote intracellular signaling activity in cell–cell and cell–extracellular matrix interactions [7]. When mouse dental papillary cells were subjected to 3D spheroid culture to compare time-dependent expression of tooth- and bone-related genes with those of cells subjected to monolayer culture, the 3D spheroid culture showed significantly higher expression of these genes [5], indicating that differentiation into mature osteoblasts is promoted in the hDPSC construct. Examination of gene expression and activity of ALP in our hDPSC construct showed that they were lower than those observed in the hDPSC sheet (Figure 5A,B). Since ALP is expressed in the early stages of osteoblast differentiation [40], lower ALP activity and gene expression indicated that differentiation into mature osteoblasts was promoted inside the hDPSC construct on day 7 of the 3D culture. Meanwhile, on day 1 of the 3D culture, the expression of all bone-related genes was significantly low (Figure 5A). We attributed this phenomenon to cell damage caused when the basal sheet was detached from the surface of the dish, as gene expressions continued to increase in a time-dependent manner after day 1 of the 3D culture.

In conclusion, an o+hDPSC construct was fabricated through monolayer culture of hDPSCs, followed by additional 3D culture in the osteogenic induction medium. The o+hDPSC construct could potentially be used as a highly useful graft material for bone tissue regeneration as it contains bone matrix proteins and calcified matrices.

## 4. Materials and Methods

### 4.1. Cell Isolation and Culture from Human Dental Pulp Tissue

All subjects provided informed consent for their inclusion before they participated in the study. The study was conducted in accordance with the Declaration of Helsinki, and the protocol was approved by the Research Ethics Committee of Tsurumi University School of Dental Medicine (Approval No. 901, 14/April/2011). Third molars were collected from 10 healthy individuals (age, 18–30 years) at Tsurumi University Dental Hospital (Yokohama, Japan). The teeth were cut at the cementum–enamel junction using a sterilized diamond bur, and the dental pulp tissue was removed from the crown and root. The tissue was digested in 3 mg/mL of type I collagenase and 4 mg/mL of dispase solution for 1 h at 37 °C and passed through a filter of 70 μm pore size (Falcon Corning, New York, NY, USA) to obtain a single-cell suspension. The isolated DPCs were cultured on 100 mm cell culture dishes (Falcon Corning) in minimum essential medium eagle, alpha modification (α-MEM: Sigma-Aldrich, St. Louis, MO, USA) supplemented with 10% fetal bovine serum (FBS; Biological Industries, Kibbutz Beit-Haemek, Israel), 100 U/100 μg penicillin/streptomycin (Life Technologies, Carlsbad, CA, USA) (basal medium), and incubated at 37 °C in 5% CO_2_ humidified atmosphere. The medium was changed twice a week. The cells reaching 70–80% confluence were harvested using 0.25% (*w*/*v*) trypsin and 1 mmol/L ethylenediaminetetraacetic acid·4 Na Solution (Wako Pure Chemical Industries Ltd., Osaka, Japan), and subcultured up to three passages. All the assays were performed using cells at the third passage.

### 4.2. Flow Cytometry Analysis

The isolated cells were cultured on 100 mm cell culture dishes until 60% confluence. We analyzed the cell surface antigen expression according to previously described methods [22]. Cells were resuspended in flow cytometry staining buffer (phosphate-buffered saline (PBS) + 2% FBS) at a concentration of 1 × 10^6^ cells/tube, followed by staining with primary specific antibodies, including phycoerythrin-conjugated CD146, APC-conjugated CD90, APC-conjugated CD73, APC-conjugated CD105, APC-conjugated CD34, APC-conjugated CD45, APC-conjugated CD14, and APC-conjugated HLA-DR (eBioscience, San Diego, CA, USA), and incubating for 30 min at 4 °C. Subclass-matched antibodies were used as negative control. The cells were then washed twice and resuspended in 400 µL of 2% FBS/PBS. Flow cytometry was performed using SH800 cell sorter (Sony, Tokyo, Japan); all the data were analyzed using FACSDiVa data acquisition software (BD Biosciences, San Jose, CA, USA). We confirmed that the isolated cells expressed mesenchymal stem cell markers, and not hematopoietic stem cell markers, and defined the isolated cells as DPSCs.

### 4.3. Preparation of hDPSC Constructs

hDPSCs were seeded into six-well culture plates (Falcon Corning) at a density of 1 × 10^5^ cells/well and cultured in basal medium for 4 weeks. The medium was changed twice a week. At 4 weeks, DPSCs formed a thick cell sheet called the basal sheet. To prepare the hDPSC constructs, basal sheets were gently detached using a cell scraper (Falcon Corning) from the plates, and cultured for another week on ultra-low cell-binding six-well plates (HydroCell, Cell Seed Europe Ltd., London, UK) with basal or osteogenic medium that comprised basal medium supplemented with 50 μg/mL of ascorbic acid (Wako Pure Chemical Industries Ltd., Osaka, Japan), 10 mM of β-glycerophosphate (Sigma-Aldrich), and 10−8 M dexamethasone (Sigma-Aldrich) for 3D culture. The medium was changed twice a week. As a control, basal sheets were cultured for 1 week in the same six-well plates with basal and osteogenic media for monolayer culture (2D).

Four types of cultures were prepared and defined as follows:

- hDPSC sheet: cultured in basal medium under 2D culture.
- o+hDPSC sheet: cultured in osteogenic medium under 2D culture.
- hDPSC construct: cultured in basal medium under 3D culture.
- o+hDPSC construct: cultured in osteogenic medium under 3D culture.

### 4.4. Characterization of the hDPSC Construct

We histologically and immunohistologically examined the hDPSC construct and hDPSC sheet. Each sample was fixed in 4% paraformaldehyde (Wako) for 24 h, dehydrated in serially degraded ethanol, and embedded in paraffin according to the conventional method. Sections were cut to 5 μm thickness, deparaffinized, and stained with hematoxylin and eosin and alizarin red S (Sigma-Aldrich, St. Louis, MO, USA). Images were captured using BZ-X700 (Keyence, Tokyo, Japan).

Bone-related proteins and cell death within the o+hDPSC construct were immunohistochemically examined; sections were deparaffinized, hydrated, and washed in PBS. After treatment with 10% normal goat serum at room temperature for 10 min, the sections were incubated with rabbit anti-osteopontin antibody (AbCam, Cambridge, UK; 1:150), rabbit anti-bone sialoprotein antibody (AbCam; 1:250), mouse anti-osteocalcin antibody (R&D Systems, Minneapolis, MN, USA; 1:250), or mouse anti-type 1 collagen antibody (AbCam; 1:150) at 4 °C overnight. After washing with PBS, OPN and BSP localization were visualized using goat anti-rabbit IgG (H + L; Alexa Flour 594; AbCam; 1:500); OCN and Col 1 were visualized using anti-mouse IgG (whole molecule)-FITC (Sigma-Aldrich; 1:32) at room temperature for 1 h. The sections were washed twice with PBS and counterstained with 4,6-diamidino-2-phenylindole (0.1 μg/mL) for 30 min to visualize the nuclei. The sections were observed using BZ-X700. For the determination of cell death in the hDPSC construct, we measured apoptosis using the In Situ Apoptosis Detection Kit (Wako Pure) and DAB immunohistochemistry substrate (Genway Biotech Inc., Sandiego, CA, USA) with minor modifications to the manufacturer’s protocol. The sections were stained with DAB solution for 5 min to determine the TUNEL-positive cells, and then counterstained with hematoxylin. The positive control sections were reacted with DNase I reaction solution before adding the terminal deoxynucleotidyl transferase reaction liquid.

Next, using five representatives of the 10 subjects, we chronologically examined the expression level of mRNA encoding *ALP*, *Col 1*, *OPN*, *BSP*, *OCN*, *Runx2*, and *osterix* in hDPSC constructs and hDPSC sheets using quantitative real-time PCR. Each sample cultured in each condition was collected at 0, 1, 3, 5, and 7 days after culturing. Briefly, total RNA was extracted from the samples every day using TRIzol reagent (Invitrogen, Carlsbad, NM, USA), and cDNA was generated from 1 µg of total RNA using SuperScript III First-Strand Synthesis System (Invitrogen). PCR amplification was performed in 25 µL of reaction mixture using 1.1 × ReadyMix PCR Master Mix (1.5 mM MgCl_2_; ABgene, Thermo Scientific, Waltham, MA, USA). PCR was performed in triplicate with SYBR Premix Ex Taq (Takara Bio Inc., Shiga, Japan) using Applied Biosystems Step One Real-Time PCR System (Applied Biosystems Inc., Carlsbad, CA, USA). Table 1 shows the conditions and primer sequences for PCR amplification. *GAPDH* was used as an internal standard for cDNA quantity and quality. The gene expression levels were calculated using the ΔΔCT method. The results were presented as fold changes of gene expression level ± standard deviation.

Each sample was cultured in each condition for 7 days and then retrieved to measure the ALP activity using an ALP assay kit (Takara Bio Inc., Shiga, Japan) and DNA Quantity Kit (Primary Cell, Hokkaido, Japan) according to the manufacturer’s instructions. First, ALP activity (units/µL) per sample was measured as follows: samples were washed thrice with PBS and lysed using 500 µL of physiological saline containing 1% Nonidet P-40. Each lysate was centrifuged at 2000 rpm for 1 min at 4 °C; then, 10 µL of the supernatant solution from each sample was added to 15 µL of *p*-nitrophenyl phosphate as the substrate. The mixture was incubated at 37 °C for 1 h, and the reaction was stopped by adding 25 µL of 0.5 N NaOH (Wako). ALP activity was determined at 405 nm absorbance using a microplate reader (Bio-Rad, California, CA, USA). Furthermore, the DNA quantity (µg/µL) in each sample was measured as follows: 10 µL of supernatant solution from each sample was combined with 200 µL of chromophore solution. The fluorescence (emission signal at 520 nm) of the mixture was detected using 490 nm excitation wavelength with a Varioskan LUX multimode microplate reader (Thermo Fisher Scientific, Yokohama, Japan). ALP activity (units/µg) was calculated as the ALP activity (units/µL) divided by the quantity (µg/µL) of DNA, in triplicate, using hDPSCs derived from five different donors.

Finally, hDPSCs derived from five different donors were stained in triplicate using alizarin red S to assess mineralization in the hDPSC constructs and hDPSC sheets. Each sample was fixed in 4% paraformaldehyde solution for 20 min, subsequently stained with 1% alizarin red S solution, incubated for 15 min at room temperature, and thoroughly washed thrice with deionized water. For quantitative measurement of alizarin red S staining, each stained sample was incubated in hydrochloric acid alcohol at room temperature for 1 h, and the absorbance of the extracted solutions was measured at 405 nm using a microplate reader.

### 4.5. Statistical Analysis

All the data were presented as the mean ± standard error. Distribution normality was assessed using Kolmogorov–Smirnov tests. The expression levels of mRNA encoding bone-related genes were analyzed using the two-way analysis of variance. The data for ALP activity and quantity of calcified matrix were analyzed using Kruskal–Wallis tests. Significances were assigned at *p* < 0.05. Analyses were performed using the SPSS Ver. 22.0 (IBM, Tokyo, Japan).

## Figures and Tables

**Table 1:** Marker and primer sequences used for real-time PCR analysis.

Primer	Forward Primer	Reverse Primer	Size (bp)
*GAPDH*	GAGTCAACGGATTTGGTCGT	TTGATTTTGGAGGGATCTCG	451
*ALP*	ACCATTCCCACGTCTTCACATTT	AGACATTCTCTCGTTCACCGCC	476
*Col 1*	CACTGGTGATGCTGGTCCTG	CGAGGTCACGGTCACGAAC	335
*OPN*	TGAAACGAGTCAGCTGGATG	TGAAATTCATGGCTGTGGAA	162
*BSP*	CAACAGCACAGAGGCAGAAA	CGTACTCCCCCTCGTATTCA	247
*OCN*	GTGCAGAGTCCAGCAAAGGT	TCAGCCAACTCGTCACAGTC	175
*Runx2*	ACTTCCTGTGCTCGGTGCT	GACGGTTATGGTCAAGGTGAA	289
*Osterix*	CCAGCCAACACTCCTACTCC	GCCTTGCCATACACTTGC	255

## Abbreviations

3D	three-dimensional
2D	monolayer culture
TEC	tissue-engineered constructs
DPSC	dental pulp stem cells
DPC	dental pulp cells
hDPSC	human dental pulp stem cells
PBS	phosphate-buffered saline
PCR	polymerase chain reaction
TUNEL	terminal deoxynucleotidyl transferase dUTP nick-end labeling
OPN	osteopontin
BSP	bone sialoprotein
OCN	osteocalcin
Col 1	type I collagen
FBS	fetal bovine serum
